# Factors Modifying Outcome After MIBG Therapy in Children With Neuroblastoma—A National Retrospective Study

**DOI:** 10.3389/fonc.2021.647361

**Published:** 2021-04-12

**Authors:** Marek Ussowicz, Aleksandra Wieczorek, Agnieszka Dłużniewska, Anna Pieczonka, Robert Dębski, Katarzyna Drabko, Jolanta Goździk, Walentyna Balwierz, Daria Handkiewicz-Junak, Jacek Wachowiak

**Affiliations:** ^1^ Department and Clinic of Pediatric Oncology, Hematology and Bone Marrow Transplantation, Wroclaw Medical University, Wrocław, Poland; ^2^ Department of Pediatric Oncology and Hematology, University Children’s Hospital, Jagiellonian University Collegium Medicum, Kraków, Poland; ^3^ Stem Cell Transplant Center, University Children’s Hospital, Department of Clinical Immunology and Transplantology, Jagiellonian University Collegium Medicum, Kraków, Poland; ^4^ Department of Pediatric Oncology, Hematology and Transplantology (EBMT CIC 641, CIBMTR Center 10797), University of Medical Sciences, Poznań, Poland; ^5^ Department of Pediatric Hematology and Oncology, Collegium Medicum, Nicolaus Copernicus University Torun, Bydgoszcz, Poland; ^6^ Department of Pediatric Hematology, Oncology and Stem Cell Transplantation, Medical University, Lublin, Poland; ^7^ Department of Nuclear Medicine and Endocrine Oncology, Maria Sklodowska-Curie National Research Institute of Oncology, Gliwice, Poland

**Keywords:** MIBG, high-dose chemotherapy, pediatric, hematopoietic stem cell transplant, treosulfan-based conditioning, busulfan and melphalan

## Abstract

**Background:**

Neuroblastoma is the most common pediatric extracranial tumor with varied prognoses, but the survival of treated refractory or relapsing patients remains poor.

**Objective:**

This analysis presents the outcomes of children with neuroblastoma undergoing MIBG therapy in Poland in 2006-2019.

**Study Design:**

A retrospective cohort of 55 patients with refractory or relapsed neuroblastoma treated with I-131 MIBG in Poland in 2006-2019 was analyzed. The endpoints were overall survival (OS), event-free survival (EFS), cumulative incidence (CI) of second cancers and CI of hypothyroidism. Survival curves were estimated using the Kaplan-Meier method and compared between the cohorts by the log-rank test. Cox modeling was adopted to estimate hazard ratios for OS and EFS, considering factors with P < 0.2.

**Results:**

Fifty-five patients with a median age of 78.4 months (range 18-193) with neuroblastoma underwent one or more (4 patients) courses of MIBG I-131 therapy. Fifteen patients were not administered chemotherapy, 3 children received standard-dose chemotherapy, and 37 patients were administered high-dose chemotherapy (HDCT) (busulfan-melphalan in 24 and treosulfan-based in 12 patients). Forty-six patients underwent stem cell transplantation, with autologous (35 patients), haploidentical (6), allogeneic (4), and syngeneic grafts (1). The median time from first MIBG therapy to SCT was 22 days. Children with relapsing tumors had inferior OS compared to those with primary resistant disease (21.2% vs 58.7%, p=0.0045). Survival was better in patients without MYCN gene amplification. MIBG therapy was never curative, except in patients further treated with HDCT with stem cell rescue irrespective of the donor type. 31 patients were referred for immune therapy after MIBG therapy, and the 5-year OS in this group was superior to the untreated children (55.2% vs 32.7%, p=0.003), but the difference in the 5-year EFS was not significant (25.6% vs 32.9%, p=ns). In 3 patients, a second malignancy was diagnosed. In 19.6% of treated children, hypothyroidism was diagnosed within 5 years after MIBG therapy.

**Conclusion:**

MIBG therapy can be incorporated into the therapeutic strategy of relapsed or resistant neuroblastoma patients as preconditioning with HDCT rather than stand-alone therapy. Follow-up is required due to the incidence of thyroid failure and risk of second cancers.

## Introduction

Neuroblastoma, which is derived from sympathoadrenal progenitor cells within the neural crest, is the most common extracranial tumor in children and shows hallmarks of neural tissue, such as the production of catecholamines and the expression of neurotrophin receptors (1). The outcome correlates with biological and clinical factors such as age, tumor stage, histology, and genetic profile, with MYCN protooncogene amplification being a prominent unfavorable risk factor (2). The prognosis varies, ranging from spontaneous regression to fatal outcomes despite aggressive therapy; in particular, in refractory or relapsing disease, survival remains very poor, with a 10-year survival probability below 15% (3). The search for therapeutics to improve the prognosis of this group of patients is warranted, but no significant break-through has been achieved in chemotherapy for the last 20 years. Because high-risk neuroblastoma patients benefit from consolidation megatherapy, many attempts have been made to improve the efficacy of this treatment. The idea of megatherapy is based on the administration of high-dose chemotherapy (HDCT) aimed at eradicating the primary malignancy, and bone marrow damage manifesting as myeloablation or myelosuppression must be subsequently managed by hematopoietic stem cell transplantation from autologous (autoSCT) sources or allogeneic (alloSCT) or haploidentical (haploSCT) donors. The results of chemotherapy and HDCT in neuroblastoma still leave a room for improvement that can be achieved by exploiting biological characteristics of malignant cells. Neural differentiation features can be used for targeted therapy in neuroblastoma and improve the clinical outcome. Ninety percent of neuroblastomas express NET, a 12-domain transmembrane protein encoded by the SLC6A2 gene with high affinity and specificity for norepinephrine and its analogs, which enables uptake and targeted therapy with I-131 radiolabeled metaiodobenzylguanidine (MIBG) (4). The indications of I-131 MIBG therapy include treatment-resistant neuroblastoma, unresectable or metastatic pheochromocytoma and paraganglioma, unresectable or metastatic carcinoid tumors, and unresectable or metastatic medullary thyroid cancer (5). The primary toxicity of I-131 MIBG therapy is hematologic, with stem cell rescue typically required with doses ≥ 15 mCi/kg (6). Based on the results of the study by Matthay et al., the MIBG dose given in megatherapy is usually 12 mCi/kg, which corresponds to a total whole body dose of 2.74 to 5.2 Gy absorbed by a patient (7, 8). The role of MIBG therapy in neuroblastoma has been confirmed, but its place in the therapeutic strategies remains unclear, and different approaches have been studied over the years. This analysis presents the results of children with neuroblastoma undergoing MIBG therapy in Poland in 2006-2019.

## Patients and Methods

A retrospective cohort of 55 patients with refractory or relapsed neuroblastoma treated with I-131 MIBG in Poland in 2006-2019 was analyzed. The patient characteristics and treatment stratifications are presented in **Table 1**.

**Table 1:** Patient characteristics.

Category		Value
**Total number**	Patients	55
	MIBG therapies	59
**Sex**	Male	32
	Female	23
**Age at therapy in months**	Median	78.4
	Range	18.3-193.2
**MYCN status**	Amplified	10
	Non-amplified	26
	Unknown	19
**Disease stage**	Stage 3	2
	Stage 4	53
**Disease status**	Relapsed	22
	Resistant	27
	Unknown	6
**Interval from diagnosis** **to MIBG therapy (in months)**	Median	15.6
	Range	5.8-133
**MIBG-activity in mCi**	Median	300
	Range	100-500
**MIBG-activity in mCi/kg**	Median	17.85
	Range	6.25-26.6
**Post-MIBG Curie score**	Median	4
	Range	0-30
**Post-MIBG Curie score**	Standard risk ≤2	25
	High risk >2	28
**Extraosseus** **MIBG uptake**	Yes	16
	No	35
**Post-MIBG chemotherapy**	None	15
	Standard dose	3
	HDCT	37
**High dose chemotherapy**	Busulfan-melfalan	24
	Treosulfan-melfalan-thiotepa	9
	Treosulfan-cyclophosphamide	3
	Fludarabine-melphalan-thiotepa	1
**Incidence of SOS/VOD** **after MIBG therapy**	Yes	5
	No	50
**Interval from MIBG therapy to SCT**	Median	22
	Range	13-103
**Stem cell transplantation**	None	11
	Autologous	37
	Allogeneic, matched sibling donor	2
	Allogeneic, matched unrelated donor	2
	Allogeneic, haploidentical donor	6
	syngeneic	1

HDCT, high-dose chemotherapy; MIBG, meta-iodobenzylguanidine; SOS/VOD, sinusoidal obstruction syndrome/veno-occlusive disease.

**Figure 1:**
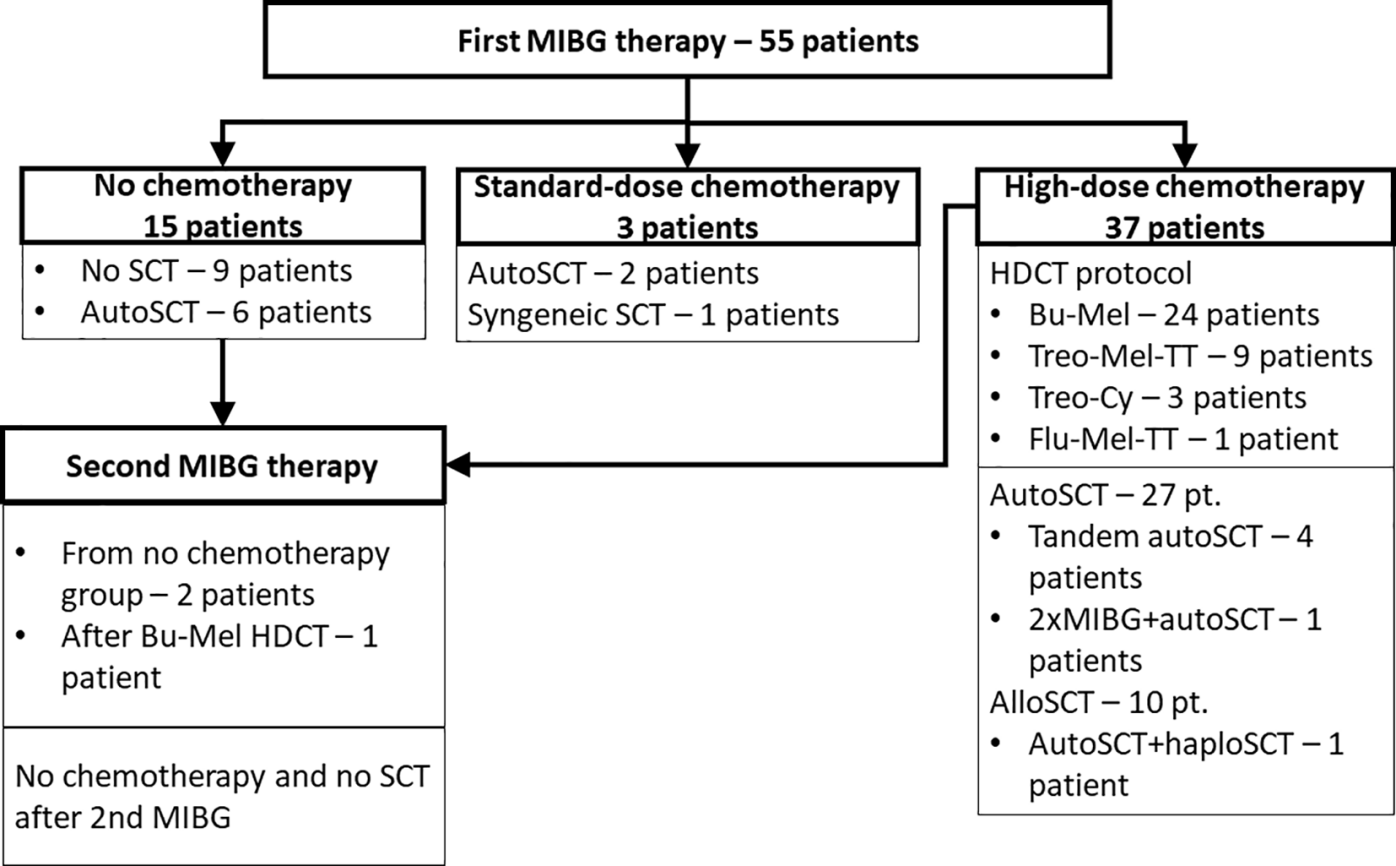
Distribution of patients treated with MIBG therapy.

The prerequirement for therapy was an avid MIBG disease within 3 months prior to therapy and unfavorable prognostic factors such as relapse or primary chemoresistance. The stage at initial diagnosis (in refractory patients) or at relapse (in relapsed children) and response at subsequent follow-up visits were assessed by a review of CT scans, bone marrow biopsies and MIBG scans and evaluated according to the INRC criteria of response (9, 10). The patients were grouped according to the response to first-line chemotherapy as patients with primary refractory (or persistent disease) or as relapsed patients who had disease recurrence at any time prior to study enrollment. The group of primary refractory patients consisted of 27 children not achieving complete remission (CR) after first-line chemotherapy, that corresponded to PR (partial response) in 21 patients, SD (stable disease) in 1 child, MR (mixed response), or PD (progressive disease) in 5 patients according to the INRC criteria. Among the 22 relapsing patients, 18 suffered from 1 relapse and 4 from multiple relapses. Autologous peripheral blood stem cells (PBSCs) were collected if bone marrow evaluation showed complete remission. The target CD34+ cell dose yield was 3 × 10^6^/kg of the recipient’s weight per autoSCT. Patients not eligible to the autologous PBSC apheresis due to poor mobilization were qualified for allogeneic stem cell transplantation from related or unrelated donor. Four children referred for palliative care were scheduled for low-dose MIBG therapy and not referred for post-therapy stem cell rescue. The decisions on palliative MIBG therapy were reached in patients with advanced and progressive malignancy on individual basis and with consent of patients’ families. The chart showing the grouping of treated patients is presented in **Figure 1**.

The administration of MIBG was performed at the central facility of Maria Sklodowska Curie Memorial Cancer Center and Institute of Oncology in Gliwice. The MIBG dose given in palliative treatment (without available stem cell rescue) was 8-12 mCi/kg and in megatherapy (patients with option of any SCT) - usually 18 mCi/kg. The dose of 131I-MIBG was adjusted to the upper limit of radioisotope use in the treating center (500 mCi). The patients received a median activity of 300 (range 100-500) mCi of MIBG. Median time from diagnosis to MIBG therapy was 15.6 months (range 5.8-133). All patients remained in radiation protective isolation for up to seven days after MIBG administration. Thyroid protection consisted daily of 1% solution of potassium iodide at dose of 2 mg/kg starting 72 hours before MIBG administration until 120 hour post therapy. Four days after MIBG injection the post therapy MIBG whole body scan was performed, and assessed with the Curie score (11).

After discharge from MIBG therapy, the patients were referred to stem cell transplantation units and treated with chemotherapy. The HDCT protocols consisted of busulfan or treosulfan backbone with additional alkylating agents. BuMel HDCT consisted of busulfan administered in 16 doses from day −7 to day −3 before autoSCT (at a cumulative dose of 16 mg/kg) and after 2009 intravenously (cumulative dose according to weight range: < 9 kg: 16 mg/kg; 9-16 kg: 19.2 mg/kg; 16-23 kg: 17.6 mg/kg; 23-34 kg: 15.2 mg/kg; >34 kg: 12.8 mg/kg), and melphalan was administered on day −1 in a single intravenous dose of 140 mg/m^2^ or in children below 12 kg at a dose of –4 mg/kg. The TreoMelTT HDCT protocol consisted of treosulfan (14 g/m2/day for 3 days), melphalan (70 mg/m2/day for 2 days), and thiotepa (2 × 5 mg/kg BW for 1 day). One of the transplant centers used a stand-alone MIBG-megatherapy in a series of patients with or without stem cell rescue as a consolidation. The parents gave their written informed consent for the treatment and analysis of clinical data. Ethical approval was waived by the local Ethics Committee of Wroclaw Medical University in view of the retrospective nature of the study, and all procedures being performed were part of routine care.

### Statistical Analysis

The endpoints were overall survival (OS), defined as the time from MIBG therapy to death or the last report from patients with no event, and event-free survival (EFS), defined as the time from MIBG therapy to progression, relapse, second malignancy or death. Survival curves were estimated using the Kaplan-Meier method and compared between the cohorts by the log-rank test. Cox modeling was adopted to estimate hazard ratios for OS and EFS, considering factors with P < 0.2. Statistical analysis and data formatting for presentation were performed with the computer software GraphPad Prism (GraphPad Software, La Jolla, CA, USA) and STATISTICA 13.3 (TIBCO Software Inc. 2017, STATISTICA, version 13, Dell, OK, USA).

## Results

**Figure 2:**
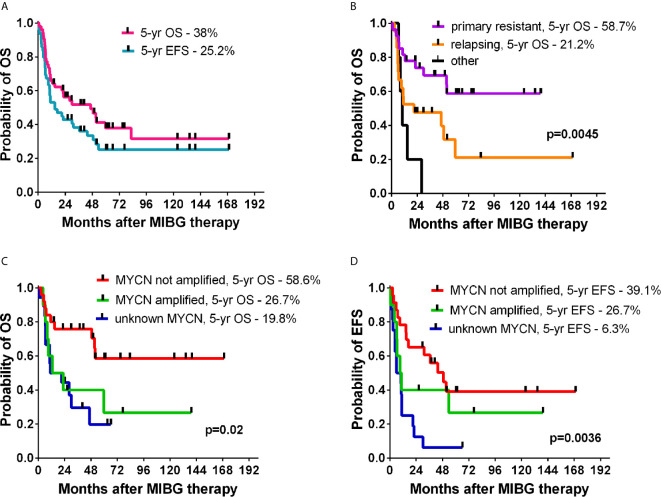
The OS and EFS probabilities of patients treated with MIBG therapy **(A)**, OS in relapsing or resistant disease **(B)**, impact of MYCN status on OS **(C)** and EFS **(D)**.

**Figure 3:**
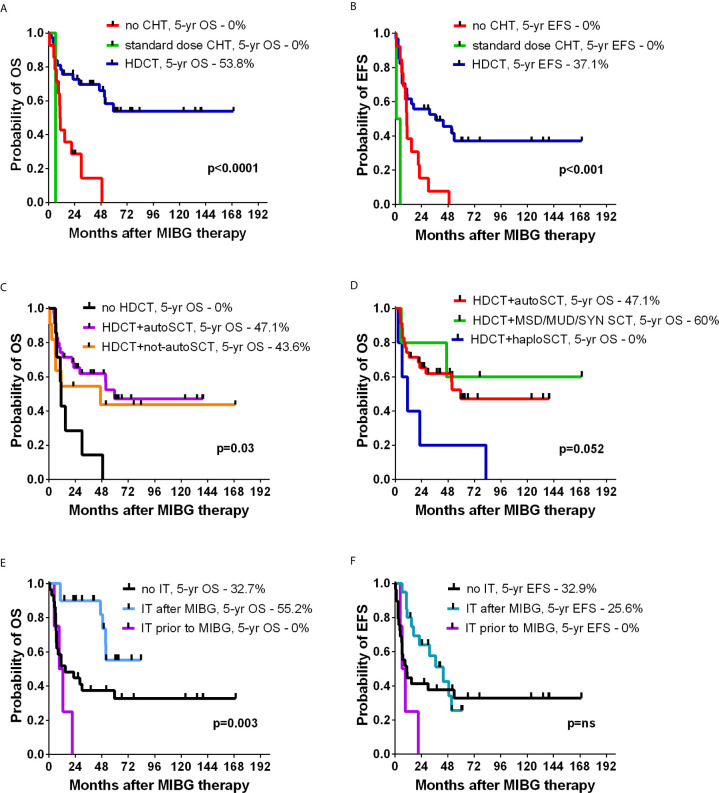
The effect of chemotherapy on OS **(A)** and EFS **(B)**. OS in patients with or without HDCT **(C)**, and impact of different donor types on OS **(D)**. The effect of immune therapy (IT) on OS **(E)**, and EFS **(F)**.

Fifty-five patients with a median age of 78.4 months (range 18-193) with neuroblastoma underwent one or more (4 patients) courses of MIBG I-131 therapy. Among 15 patients without chemotherapy, 6 required SCT due to prolonged cytopenia, and 1 died due to fulminant disease progression. Standard dose chemotherapy was given in 3 patients: VP-Carbo/etoposide-carboplatin in 1, and CADO/cyclophosphamide, doxorubicin and vincristine in 2 children. In 37 patients, HDCT was performed (BuMel in 24 patients, treosulfan-based therapy in 12 patients, and other in 1 patient). The grafting material was autologous in 35 children, haploidentical from the parent in 6 children, allogeneic in 4 children, and syngeneic in one child. The median time from first MIBG therapy to SCT was 22 days (range 13-103). Detailed survival results are presented in **Table 2**. In the whole group, the probability of 5-year OS was 38%, and the probability of 5-year EFS was 25.2% (**Figure 2A**). Patients with relapsing disease had inferior survival compared with those with primary resistant disease (5-year OS 15.2% vs 58.7%, p=0.001) (**Figure 2B**). The MYCN status affected survival, which was superior in patients without amplification (5-year OS 58.6% and 5-year EFS 39.1%, **Figures 2C, D**). MIBG therapy was never curative (5-year OS and 5-year EFS 0%), except in patients further treated with HDCT with stem cell rescue irrespective of their donor type (p<0.001) (**Figures 3A, B**). The HDCT protocol was based on busulfan or treosulfan in almost all patients, and the 5-year OS and 5-year EFS were not different between the groups. Sinusoidal obstruction syndrome/veno-occlusive disease (SOS/VOD) was diagnosed in 5 of 24 patients (20.8%) in the BuMel HDCT group, but not after any other chemotherapy protocols.

**Table 2:** Survival after MIBG therapy.

		Number of patients	5 year OS	log rank p	5 year EFS	log rank p
All patients		55	38%	n/a	25.2%	n/a
Sex	Male	32	51.9%	ns	31.3%	ns
	Female	23	29%		19.7%	
Disease status at MIBG therapy	Resistant	27	58.7%	p=0.0045	45%	p=0.0003
	Relapsed	22	21.2%		11.4%	
	Not specified	6	0%		0%	
MYCN status	Amplified	10	26.7%	p=0.02	26.7%	p=0.0036
	Not amplified	26	58.6%		39.1%	
	Unknown	19	19.8%		6.3%	
Post-MIBG Curie score	≤2	25	42.2%	ns	32.1%	ns
	>2	28	36.7%		25.7%	
Extraosseus MIBG uptake	Yes	17	41.7%	ns	30%	ns
	No	36	36.8%		27.4%	
Chemotherapy	No	15	0%	p<0.0001	0%	p<0.001
	Standard	3	0%		0%	
	HDCT	37	53.8%		37.1%	
HDCT	Busulfan	24	43.9%	ns	31%	ns
	Treosulfan	12	66.7%		50%	
SCT	No	9	0%	p=0.03	0%	ns
	Auto	35	47.1%		31.2%	
	Allo/haplo/syn	11	43.6%		27.3%	
Anti-GD2 immunotherapy	No	20	32.7%	p=0.003	32.9%	ns
	Yes, after MIBG	31	55.2%		25.6%	
	Yes, prior to MIBG	4	0		0	

Allo, allogeneic; auto, autologous; HDCT, high-dose chemotherapy; n/a, not applicable; ns, not significant; SCT, stem cell transplantation; syn, syngeneic.

In the analyzed group, 10 patients were transplanted from allogeneic donors due to a lack of sufficient stem cell harvest or upfront strategy for haploidentical transplantation in relapsing tumors. HDCT resulted in better outcomes than non-HDCT, and the results of autologous SCT were similar to those of non-autologous grafts (**Figure 3C**). However, among different types of donors, haploidentical transplantations showed a trend toward worse survival, and no long-term survivors were observed after haploSCT (**Figure 3D**).

In 53 patients a post-therapy MIBG whole-body scan was performed, and in 40 cases the uptake was detected, with 28 patients showing the Curie score above 2 and in 17 the extraosseus foci were observed. In 13 patients, the post-therapy scan did not reveal MIBG uptake, and 9 of these patients showed subsequent relapse and 7 died of disease.

Among 55 patients, 31 were referred for immune therapy after MIBG therapy, and the 5-year OS in this group was superior to the untreated children (55.2% vs 32.7%, p=0.003, **Figure 3E**), but the difference in the 5-year EFS was not statistically significant (25.6% vs 32.9%, p=ns, **Figure 3F**).

In the Cox multivariate analysis, autoSCT and post-MIBG immune therapy emerged as the only factors significantly associated with improved OS (hazard ratio 0.58, 95% CL 0.17-1.93; P=0.04; and hazard ratio 0.08, 95% CL 0.0-0.72; P=0.02). The other factors were not significantly associated with either OS or EFS.

### Adverse Effects and Second Malignancies

**Figure 4:**
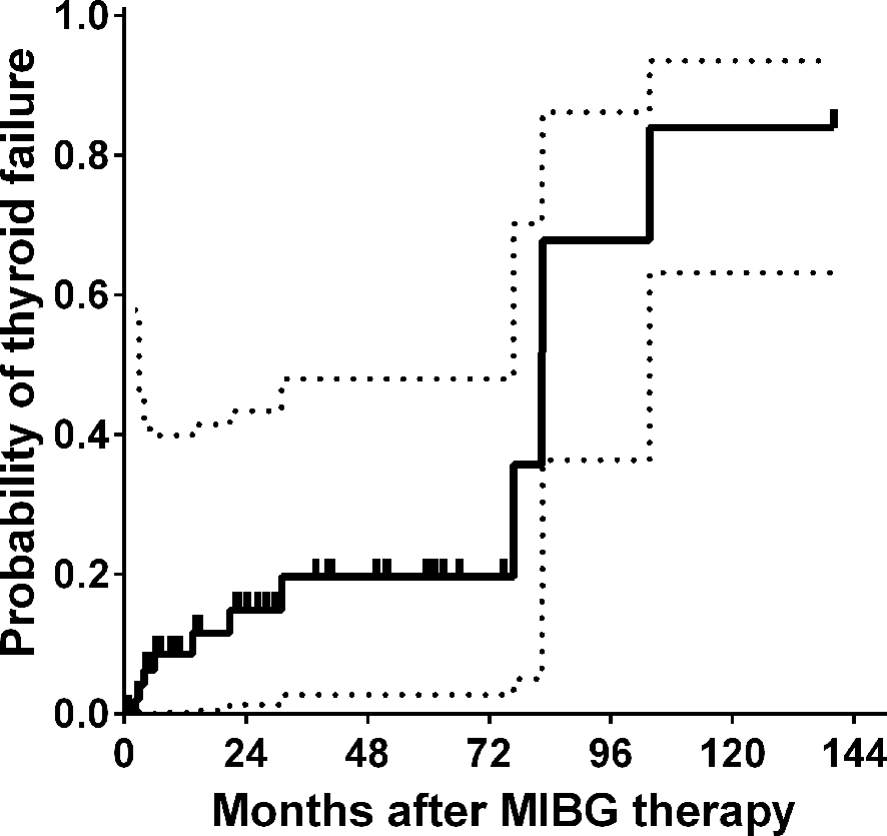
Probability of thyroid failure after MIBG therapy.

In 19.6% of treated children, hypothyroidism was diagnosed within 5 years of MIBG therapy (**Figure 4**). In 3 patients, a second malignancy was diagnosed, which corresponded to a 5-year cumulative incidence of 10.9%. In a girl coded as UPN 26, diffuse large B-cell lymphoma of the brain was diagnosed, and the child died as a consequence of it. In patients UPN 32 and UPN 33, myelodysplastic syndrome with excess blasts (MDS-EB) was diagnosed. Both patients with MDS-EB were transplanted from matched donors and are alive and well.

## Discussion

I-131 MIBG is one of the successful examples of theranostic radiopharmaceuticals but requires specialized facilities with appropriate personnel, radiation safety equipment, procedures available for waste handling and disposal, the handling of contamination, monitoring personnel for accidental contamination and the controlling contamination spread, which are limiting factors of the widespread use of this therapy (12, 13). Despite the high number of neuroblastoma patients treated with MIBG-megatherapy, the mean tumor response rate in 25 studies was estimated at 32%, but the meta-analysis by Wilson et al. showed high heterogeneity of analyzed studies and a lack of randomized controlled trials (14). The 38% 5-year OS in our whole study group combined the better outcome (58.7%) of patients with primary resistant disease and the worse outcome (15.2%) of patients with relapsing disease, which is consistent with that in other studies. Our study was limited by the small number and heterogeneously treated group of patients over a long period of time in different centers, but worse outcomes in relapsing patients were also observed in other studies. According to the study by Zhou et al. who reviewed the retrospective cohort of 218 patients treated with (131)I-MIBG between 1996 and 2014, the probability of 24-month OS was 47.0%, and for refractory patients was significantly higher at 65.3%, compared to 38.7% for relapsed patients (p<0.001) (15). In our group the respective 24-month OS results were 56.2% (in the whole group), 73.7% in refractory, and 38.5% in relapsing patients, which suggests similar early outcome despite different treatment protocols, but deteriorating survival after 5 years emphasizes incomplete and impermanent responses.

Matthay et al. reported that stand-alone MIBG therapy showed hematological toxicities (thrombocytopenia and/or neutropenia), resulting in HSCT in 33% of treated children (6). The response rate was significantly higher for patients with disease limited either to bone and bone marrow or to soft tissue (compared with patients with both) for those with fewer than three prior treatment regimens and for patients older than 12 years; OS was 49% at 1 year and 29% at 2 years, but EFS was only 18% at 1 year (6). The influence of skeletal or extraskeletal disease was not confirmed in our study, and the post-therapy whole body scan result was not associated with differences in survival. This effect might be a consequence of maturation of neuroblastoma cells in the persisting skeletal MIBG avid lesions, but no surviving patients in our study underwent the bone biopsy.

In our study, eleven of 15 children treated with stand-alone MIBG-therapy were not treated with intention of palliative care, and all of them died. Long-term survivors were noted only in the arm combining MIBG therapy with HDCT, which suggests, that the survival benefit is unlikely in stand-alone MIBG treatment. However, it must be noted that the HDCT protocol choice and time of administration require prospective study.

The combination of MIBG therapy with HDCT has raised the issue of therapy tolerance and excessive toxicities on many occasions, and different approaches have been suggested. Among posttransplant risks for neuroblastoma patients, the incidence of SOS/VOD is high. In our study, SOS/VOD incidence of 20.8% was similar to the 22% observed in the patients treated with BuMel HDCT alone (16). In a study by Yanik et al., the combination of MIBG megatherapy administered on day -21, with CEM given days -7 to -4, and SCT given on day 0 was evaluated (17). The study showed that the SOS/VOD incidence was 12%, which is similar to the 9% reported for the CEM protocol alone (16). A study by Miano showed an increased risk of toxicities induced by the addition of MIBG therapy 7 days prior to BuMel (11 patients) or BuMel with thiotepa (4 patients) HDCT but showed the feasibility of such a combination in children with neuroblastoma (18). A recent study by Giardino et al. showed that MIBG therapy administered shortly before BuMel HDCT was well tolerated, and the SOS/VOD incidence was 7.1% (19). The cumulative incidence of hypothyroidism was 31.1% in this study, the three-year and five-year rates of the cumulative risk of progression/relapse were 64% and 73%, respectively, and MYCN amplification emerged as the only risk factor significantly associated with OS (HR, 3.58; P = 0.041). The concern of toxicities resulted in the strategy of delayed HDCT after MIBG therapy and was studied by French et al., who administered MIBG therapy with first autoSCT and 6-8 weeks later administered BuMel with second autoSCT (20). The straightforward combination (MIBG therapy-HDCT-1^st^ autoSCT) in our study was used in 32/37 patients, and due to the low number of tandem transplantations (MIBG therapy-1^st^ autoSCT-HDCT-2^nd^ autoSCT), a detailed analysis of this group was not performed. The strategy of combining MIBG therapy with BuMel as tandem transplantation is a safer approach, as demonstrated by the French group, although it requires double the amount of collected stem cells (21). The strategy of tandem transplantation reduces the chemotherapy dose intensity, which may be associated with worse efficacy, as MIBG stand-alone therapy effectiveness is less potent than combination with HDCT. In addition, some reports have suggested that the toxicities after combination therapy are manageable and do not significantly differ from megachemotherapy alone. Preconditioning with MIBG is feasible with treosulfan-melphalan-thiotepa HDCT, and the 2-week interval between both therapies was not associated with excessive toxicities (22). The combination of MIBG preconditioning with megachemotherapy can reduce the need for a high stem cell yield in pretreated patients, which reduces the delay due to ineffective stem cell mobilizations. Another strategy for patients with inadequate autologous stem cell harvest is the choice of allogeneic donor, but this option is not actively encouraged today due to limited evidence for graft versus tumor effects against neuroblastoma and the availability of plerixafor, which improves the autologous stem cell yield (23, 24). Indeed, no benefit of haploSCT was observed in our study despite the promising results reported by Illhardt et al. (25).

According to the SIOPEN study group, the recommended HDCT protocol in neuroblastoma is BuMel (16). Recently, treosulfan has been viewed as a promising option, especially because of the data supporting the *in vitro* activity of treosulfan in neuroblastoma lines (26). In our study, 12 children were treated with treosulfan-based HDCT protocols and achieved solid survival rates (5-year OS – 66% and 5-year EFS – 50%). This observation can support the planning of treosulfan studies in neuroblastoma patients who are not eligible for busulfan-based HDCT.

The post MIBG therapy whole body scan in high-risk neuroblastoma patients may reveal hidden mIBG-avid lesions, which are under the level of detection by diagnostic 123I-MIBG images (27–29). The fact, that in 13 patients the MIBG uptake was absent can be explained by a time interval between the qualification and administration of the therapy, during which patients received chemotherapy. In the analyzed group, skeletal or extraskeletal uptake and Curie scoring were not associated with different survival, but it can be explained by the fact, that Curie score has been validated to assess the postinduction response in neuroblastoma patients, and not in the megatherapy settings (30).

Immune therapy with dinutuximab or dinutuximab beta is associated with the most important recent treatment improvement in neuroblastoma, but it has not been studied in patients undergoing the MIBG therapy (31, 32). Through 2014 immunotherapy with dinutuximab beta was not available as a therapeutic option for Polish children and those who received it did so abroad within the LTI (Long-Term Infusion study, EudraCT 2009-018077-31). In children referred for immune therapy an overall survival advantage was observed, but not the probability of 5 year EFS. Interestingly, four of 15 patients not receiving the chemotherapy were treated with immune therapy after MIBG (and 3 - before), and all of them died. The impact of immunotherapy after MIBG therapy should be studied in a prospective trial in a more homogenous cohort, and from our data no further conclusions can be drawn.

The prevention and early diagnosis of long-term sequelae are now important issues in pediatric oncology. Thyroid protection is an issue that warrants attention in patients treated with MIBG megatherapy. Clement et al. studied long-term survivors after MIBG therapy and showed that at a median follow-up of 9.0 years, the incidence of thyroid disorder was 50% (12/24 patients); among them, 5 were diagnosed with a thyroid nodule, and 1 patient was subsequently diagnosed with differentiated thyroid carcinoma (33). At 5 years after MIBG therapy, we observed a cumulative incidence of thyroid failure of 19%, and among the long-term survivors, the incidence could increase to over 80%, but the number of observed patients was very low. The risk of second malignancies also needs to be considered in patients after MIBG therapy. The review of 644 patients treated with MIBG therapy performed by Huibregtse et al. showed a second malignant neoplasm cumulative incidence of 7.6% and 14.3% at 5 and 10 years from the first therapy, with myelodysplastic syndromes/therapy-related acute myeloid leukemia being most common (10/19 patients), followed by solid tumors (inflammatory myofibroblastic tumors, bone and soft tissue sarcomas, and thyroid cancers) (34). In our group, 3 patients developed second malignancies, but it is not possible to associate the incidence of MDS with either MIBG therapy or HDCT because the patients were treated with intensive chemotherapy before referral for HDCT. In the third case, the diagnosis of lymphoma was associated with T-cell–depleted haploidentical transplantation and subsequent EBV replication.

## Conclusion

Prospective, randomized controlled trials are needed to optimize therapeutic strategies incorporating MIBG therapy in patients with neuroblastoma. Currently, 2 ongoing studies are focusing on MIBG, the upfront Children Oncology Group NCT03126916 study and SIOPEN VERITAS study (NCT03165292). MIBG therapy can be incorporated into the therapeutic strategy of relapsed or resistant neuroblastoma patients, but our analysis suggests that the advantage is achieved by using MIBG therapy as preconditioning with HDCT rather than stand-alone therapy.

Due to the lack of systematic publications on the effects of immune therapy in patients undergoing MIBG therapy, such analysis is necessary in uniform cohorts of treated children.

According to our observations, MIBG therapy patients require endocrinological follow-up due to the incidence of thyroid gland failure and the risk of second cancers.

## Data Availability Statement

The data analyzed in this study is subject to the following licenses/restrictions: available on request. Requests to access these datasets should be directed to ussowicz@o2.pl.

## Ethics Statement

The parents gave their written informed consent for the treatment and analysis of clinical data. Ethical approval was waived by the local Ethics Committee of Wroclaw Medical University in view of the retrospective nature of the study, and all procedures being performed were part of routine care.

## Author Contributions

Concept, data collection, analysis, writing, and final draft: MU. Data collection, patient care, and manuscript acceptance: AW, AD, AP, RD, KD, JG, WB, DH-J, and JW. All authors contributed to the article and approved the submitted version.

## Funding

Source of support- Wroclaw Medical University statutory grant ST.C200.18.013 and SUB.C200.21.058.

## Conflict of Interest

The authors declare that the research was conducted in the absence of any commercial or financial relationships that could be construed as a potential conflict of interest.
